# Combination Preemptive Peripheral Nerve Block in Limb Surgery. A Prospective Study

**DOI:** 10.3390/medicina56080388

**Published:** 2020-08-03

**Authors:** I-Cheng Lu, Shu-Hung Huang, David Vi Lu, Chun Dan Hsu, Sheng Hua Wu

**Affiliations:** 1 Department of Anesthesiology, College of Medicine, Kaohsiung Medical University, Kaohsiung 807, Taiwan; u9251112@gmail.com; 2 Department of Anesthesiology, Kaohsiung Medical University Hospital, Kaohsiung 807, Taiwan; 3 Department of Anesthesiology, Kaohsiung Municipal Siaogang Hospital, Kaohsiung 812, Taiwan; david1628@gmail.com (D.V.L.); mstg1103@gmail.com (C.D.H.); 4 Department of Surgery, College of Medicine, Kaohsiung Medical University, Kaohsiung 807, Taiwan; huangsh63@gmail.com; 5 Department of Surgery, Kaohsiung Medical University Hospital, Kaohsiung 807, Taiwan; 6 Department of Anesthesiology, Kaohsiung Municipal Ta-Tung Hospital, Kaohsiung 801, Taiwan

**Keywords:** peripheral nerve block, limb surgery, pain control

## Abstract

*Background and objectives*: Patients often suffer from moderate to severe pain during the early recovery period in orthopedic surgery. We investigated the impact of a single-shot preoperative peripheral nerve block (PNB) on post-anesthesia recovery parameters and interleukin (IL)-6 level during limb surgery. *Materials and Methods:* A prospective randomized controlled study was conducted, and patients scheduled for limb surgery were recruited. Sixty patients were randomly assigned to either the PNB group or control group, who received morphine as a primary analgesic. The peak verbal numeric rating scale (NRS) score in the post-anesthesia care unit (PACU) was evaluated as a primary outcome. We also recorded rescue analgesics requirement and wake-up time from anesthesia in the PACU. In addition, the change of plasma IL-6 level after incision was measured. *Results*: Fifty-two patients completed the study, 27 and 25 cases in the PNB and control group, respectively. Preemptive PNB significantly reduced peak NRS score in the PACU compared to control group. Lower rescue analgesics requirement and rapid wake-up from anesthesia were also noted in PNB group. The IL-6 concentration increased less in the PNB group at 2 h after incision. *Conclusions*: Preemptive PNB attenuates IL-6 expression 2 h after incision and improves pain management in the PACU. PNB was considered as an essential part of pain management in limb surgery.

## 1. Introduction

Poor pain control after surgery may lead to chronic post-surgical pain and some patients even experienced persistent pain over three months postoperatively [1,2,3,4,5]. Adequate pain control impacts on early ambulation [6,7] and avoidance of long-term complications [8,9]. Opioid prescriptions for managing surgical pain are common. However, these may raise some dose-dependent adverse effects, such as nausea, vomiting, drowsiness, respiratory depression, and possible lead to chronic opioid abuse [10]. High-dose opioids also have a potential role in facilitating pain sensitization and the development of persistent postoperative pain [11]. Research in patients undergoing surgery on extremities mentioned that opioids alone are associated with less satisfaction with pain relief and suggest combined alternative analgesic methods to decrease systemic opioid use [12].

Furthermore, patients who are elderly or with co-morbidities constitute a high proportion of extremity surgery incidences. Surgical trauma is a significant stressor for patients and evokes an inflammatory cascade following the release of cytokines [13]. Inflammatory responses to surgery include an increase in proinflammatory cytokines and a decrease in anti-inflammatory cytokines [14,15]. These substances can exert harmful generalized responses and perhaps lead to multiple organs dysfunction [14,16]. It is important to use appropriate modalities to manage nociception intraoperatively, attenuate surgical-induced stress response, decrease hemodynamic fluctuation during surgery, and improve pain control after surgery. In a multiplex cytokine survey in total hip replacement, systemic level of interleukin 6 (IL-6) significantly increased during the postoperative period [17]. In general, IL-6 concentration increases within 30–60 min after incision and the production of IL-6 is significantly increased after 2 to 4 h [18].

Employing opioid free anesthesia with alternative analgesic drugs is important for preventing perioperative complications [19]. Several approaches were developed to decrease opioid consumption, including preemptive analgesia to avoid precipitate acute noxious inflammatory reactions and central sensitization [20,21]. Preemptive analgesic regimens were reported for perioperative pain relief under general anesthesia, such as parecoxib [22,23], acetaminophen [24], pregabalin [25] and ketamine [26]. Accumulating evidence has shown that peripheral nerve blocks (PNB) improve acute pain management either in the emergency department [27,28] or during the postoperative period [29,30]. However, the impact of PNB on post-anesthesia recovery parameters and the change of serum IL-6 concentration in response to extremity surgery has not been fully investigated. In the study, we assess the numeric rating scale (NRS) score, rescue analgesics required, and wake-up time in the post-anesthesia care unit (PACU), as well as the change of plasma IL-6 concentration after incision in limb surgery.

## 2. Materials and Methods

The study was approved by the hospital Ethics Committee (KMUHIRB-F(I)-2017-0007) in February 2017. The study was also registered at ClinicalTrails.gov (NCT03913650). Written informed consent was obtained from each subject before anesthesia. Patients were recruited if they were mentally oriented, American society of anesthesiologists class I-III and undergoing extremity procedures. Patients with known drug or alcohol abuse, chronic pain with analgesics, previous adverse reactions to local anesthetics, opioids, or non-steroidal anti-inflammatory drugs (NSAID), and bleeding tendency were excluded from the study. In addition, operation time over 3 h, inadequate blood samples, and loss of postoperative follow-up were also excluded. All patients were assigned into two groups randomly by a computer-generated table. Patients who received single shot preemptive PNB following general anesthesia were allocated to Group N and those did not receive PNB were allocated to Group C (control group).

### 2.1. Anesthetic Protocol

All patients received standard monitor including electrocardiography, pulsed oximetry, and non-invasive blood pressure monitors. Anesthesia was induced by intravenous lidocaine (1 mg/kg), propofol (2 mg/kg) and fentanyl (1 mcg/kg). A laryngeal mask airway was placed after adequate anesthetic depth was achieved. Target-controlled infusion of propofol with effect concentration (2–5%) was used to maintain general anesthesia. Patients in Group N received real-time ultrasound guidance PNB with 0.25% bupivacaine via brachial plexus, femoral, lateral femoral cutaneous, or sciatic nerve according to the surgical sites before incision. Two experienced anesthesiologists (over 100 injections) performed all ultrasound-guided PNB injections with a linear ultrasound transducer (6–10 MHz). In both groups, anesthetic adjuvants with inhalation agent sevoflurane were applied to alleviate noxious stimulation when necessary. After surgery, all patients were transferred to the PACU for further care. Morphine was administered for primary postoperative pain medication in the PACU. Intravenous morphine 0.05 mg/kg was prescribed to the Group C for postoperative pain and both groups if NRS greater than 3 in the PACU. Rescue NSAID (parecoxib or ketorolac) could be given if poor response or intolerance to morphine was noted. Patients were allowed to discharge when they met the PACU discharge criteria (modified Aldrete score of 9–10) [31] and returned to the ward.

### 2.2. Interleukin-6 (IL-6) Concentration Measurement

Peripheral blood samples were collected and stored in ethylenediaminetetraacetic acid (EDTA) tubes. The blood samples were centrifuged at 3000 rpm for 10 min to obtain plasma. The plasma was stored at −80 °C until analysis. IL-6 concentrations were analyzed by commercially available Enzyme-Linked Immunosorbent Assay (ELISA) kit (10 pg/mL).

### 2.3. Outcome Measures

The primary outcome was the peak verbal NRS pain score ranging from 0 (no pain) to 10 (worst pain imaginable) [32] in the PACU, and pain intensity was measured at four time points (awake, 15 and 30 min after wake-up, and time of discharge from PACU). We also recorded wake-up time, total morphine consumption, and incidence of NSAID supplement for poor pain control with morphine during the period of PACU. Wake-up time was defined as the period between cessation of anesthetic delivery to response the command “open your eyes” [33].

The change of proinflammatory cytokine IL-6 level after incision was evaluated in each group. For IL-6 measurement, the blood samples were collected at three time points (preoperatively, 1 h post-incision and 2 h post-incision). We also evaluate perioperative hemodynamic variation. The perioperative hemodynamic parameters, including mean arterial pressure (MAP) and heart rate (HR) were recorded at six time points (T0: before anesthesia, T1: after LMA insertion, T2: at the time of skin incision, T3: 1 h after incision, T4: at the end of surgery, T5: peak value during PACU period).

Adverse events 1 day after surgery were followed, including dizziness, nausea/vomiting pruritis, respiration depression, and toxicity of local anesthetic. Patient satisfaction was measured by a four points scale; 1 = poor, 2 = fair, 3 = good, and 4 = excellent. Motor and sensory block were also evaluated to detect any possible neurological deficits, as previously described [34]. Sensory blockade was scored as 0 = no sensory block, 1 = loss to cold sensation and no loss to pinprick sensation, and 2 = loss to both cold and pinprick sensation. Motor blockade was scored as 0 = no motor block, 1 = partial block, and 2 = complete motor block.

We assumed that ultrasound guided PNB would produce a NRS decrease in the pain intensity of 2 as a clinical significant improvement. Sixteen patients in each group were calculated to identify this difference (with a type I error of 0.05 and power of 0.8, including a correction for a possible 10% loss to follow-up). The trial enrolled 30 cases in each group to ensure the power. Statistical analysis of continuous variables between groups was carried out by the Student *t*-test. Intragroup statistical analysis of continuous variables were compared using the paired *t*-test. Categorical nominal variables were analyzed with the Fisher exact test. All statistical tests were 2-tailed, and *p* values < 0.05 were considered statistically significant. Continuous data were expressed as mean ± standard deviation (mean ± SD). Categorical data were expressed as *n* (%). The entire analysis was performed using the statistical Package for the Social Sciences (SPSS, version 14.0)

## 3. Results

**Figure 1:**
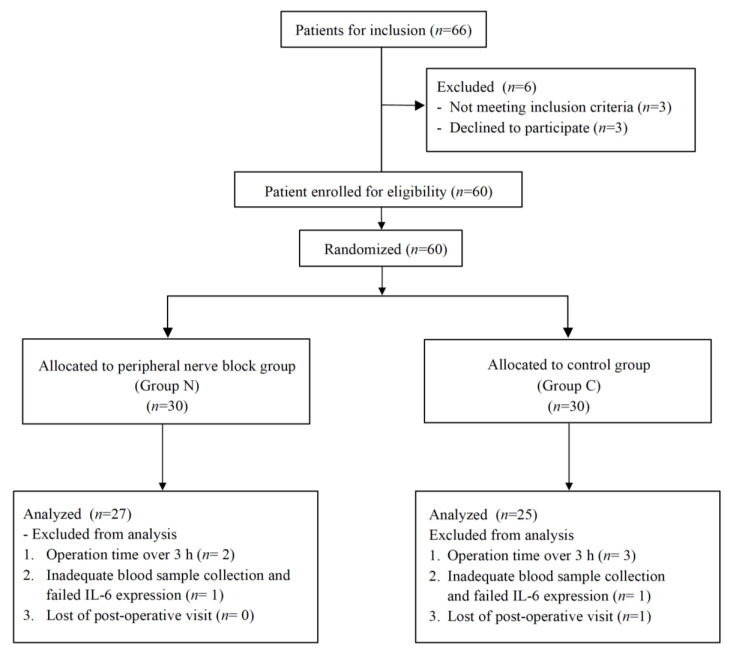
Flowchart of the study. Sixty patients scheduled for elective extremity surgery were recruited. The flowchart showed 52 patients completed the study, with 27 cases in Group N and 25 cases in Group C.

Sixty patients scheduled for elective extremity surgery were recruited from May 2017 to December 2018. The flowchart of the study showed 52 patients completed the study, with 27 cases in Group N and 25 cases in Group C (Figure 1). Five patients were excluded from our study due to actual operation time being over 3 h. Two patients that did not have adequate blood samples were also excluded. One patient was discharged within 24 h after surgery and was lost at the postoperative visit. The two groups had comparable patient characteristics (Table 1). In Group N, 7 brachial plexus blocks, 11 femoral nerve blocks, 6 lateral femoral cutaneous blocks, and 3 sciatic nerve blocks were performed. In Group C, 8 brachial plexus blocks, 10 femoral nerve blocks, 4 lateral femoral cutaneous blocks, and 3 sciatic nerve blocks were performed.

The primary outcome of peak verbal NRS pain score during PACU was significantly lower in Group N than in Group C (1.15 ± 1.81, 5.40 ± 2.48 respectively, *p* = 0.001) (Table 2). Total morphine consumption was significantly lower in the Group N than Group C (1.85 ± 2.04 mg and 6.44 ± 3.22 mg respectively, *p* = 0.001). Fewer patients required rescue NSAID analgesics in Group N than in Group C (11.1% vs. 56%, *p* = 0.003) in the PACU. Less wake-up time was noted in Group N (*p* = 0.025).

**Figure 2:**
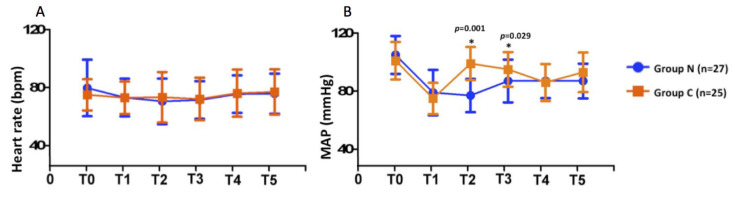
Perioperative hemodynamic changes at six time points. (**A**) Time course of perioperative heart rate changes. (**B**) Time course of perioperative mean arterial pressure changes. T0: before anesthesia induction, T1: after anesthesia induction and laryngeal mask airway insertion, T2: time of incision, T3: 1 h after incision, T4: end of surgery, T5: at the post-anesthetic care unit. * *p* values < 0.05.

**Figure 3:**
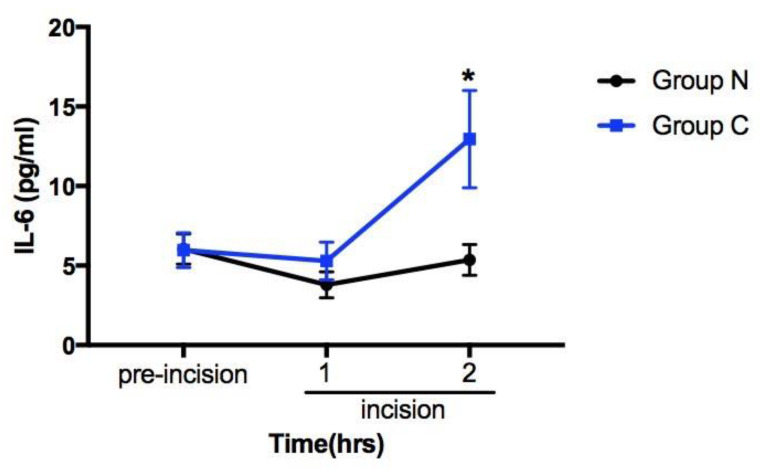
Changes of serum interleukin 6 (IL-6) concentration at three time points, including pre-incision, incision 1 h, and incision 2 h. * *p* values < 0.05.

The impact of PNB on perioperative hemodynamic changes were illustrated in Figure 2. There was no significant difference in HR between the two groups. MAP was significantly higher in Group C at the time of skin incision (*p* < 0.001) and 1 h after incision (*p* < 0.05). Less MAP fluctuation in response to surgical manipulation was observed in Group N within 1 after incision. Plasma IL-6 concentrations between two groups were similar before surgery and 1 h after the skin incision. Lower circulating IL-6 concentration was noted in Group N than in Group C at 2 h after skin incision (5.36 ± 5.01 pg/mL vs. 12.95 ± 15.32 pg/mL, *p* = 0.027; Table 3 and Figure 3).

Incidence of adverse effects and patient satisfaction on 1 day after surgery was summarized in Table 4. The experience of postoperative dizziness was reduced in Group N (7.4% vs. 32%, *p* = 0.024). Patient satisfaction did not differ significantly between groups. Residual motor and sensory blockade were exanimated on the first day. One patient experienced mild sensory impairment and four patients felt mild limb weakness on the postoperative day 1. For blinded analysis, the investigator also measured the sensory and motor blockade of control patients. No obvious significant difference in sensory and motor between two groups.

## 4. Discussion

The major finding of our study is that preoperative single-injection PNB as a part of multimodal regimen in extremity surgery attenuates postoperative pain intensity, reduces total morphine consumption, and decreases rescue NASID analgesics in the PACU as well as provides rapid wake-up from general anesthesia. Less blood pressure variation in response to surgical stimuli was found in Group N. Group N also showed less increase in proinflammatory cytokine IL-6 level at 2 h after incision. In addition, less postoperative dizziness on 1 day after surgery was found in Group N. These findings suggest preemptive PNB could play a role as a valuable analgesic regimen upon the surgical stress response in patients undergoing extremity surgery.

In extremity procedures, patients usually suffer from moderate to severe postoperative pain. As is known, opioids are widely used for postoperative pain control. However, several adverse events are related to traditional opioid analgesics and limit its use [35]. These side effects appear to occur in a dose-dependent fashion. Many analgesic adjuncts are available to improve postoperative pain control and limit opioid consumption, such as acetaminophen and NSAIDs. PNB is an alternative that offers enhanced postoperative pain control as well as reduced side effects. PNB also reduces length of hospital stay and readmissions in total knee arthroplasty [36]. PNB is not uncommonly employed as a part of general anesthesia in various surgeries, including extremity surgery [37,38,39,40,41]. In our hospital, PNB injection under general anesthesia, performed by anesthesiologists with expertise in ultrasound, is widely used for extremity procedures, especially in cases that cannot accommodate to nerve block or neuraxial anesthesia alone. One of the most important options is to establish a secure airway and provide sufficient ventilation. Furthermore, some patients experienced discomfort during PNB injection despite being under conscious sedation [42]. A previous study suggested brachial plexus block under general anesthesia had similar success and complication rates with non-anesthetized patients [38].

Surgery often induces stressful response by sending impulses from the incision site to the hypothalamus [43]. Stress hormone cortisol and catecholamines, as well as proinflammatory cytokines, are released in the perioperative period. Consequently, perioperative hemodynamic instability is related to activation of the sympathetic stimulation by surgical stress and pain associated with cardiovascular adverse events in cardiac or non-cardiac surgery [44,45]. Some methods were applied to modulate the surgical stress. Preemptive gabapentin attenuated IL-6 production in total knee arthroplasty on postoperative day 1 [46]. Elderly patients undergoing colorectal laparoscopic surgery experienced significant elevation of C-reactive protein and IL-6 after surgery [47]. Enhanced recovery after surgery protocols combined with PNB improved surgical stress response. Transversus abdominis plane block was postulated to attenuate the production of IL-6 in patients of advanced age undergoing laparoscopic rectal cancer surgery, and femoral nerve block reduced the expression of TNF-α in elderly patients with a hip fracture [48,49]. Previous study also showed that preoperative ultrasound-guided rectus sheath block inhibited the increase of IL-6 in patients undergoing transabdominal gynecological surgery [50]. In our study, MAP increased less within 1 h of incision and IL-6 concentration was elevated less at 2 h after incision in patients receiving PNB. The above findings indicated that combined with PNB, this might alleviate stress response in patients undergoing extremity procedures.

Neurological injury is an infrequent complication following PNB. Studies have reported the incidence is around 0.5 to 10% [51,52,53,54], with most patients complaining about numbness or limb weakness for a few days to weeks. Long-term neuropathy directly attributed to nerve block is rare [55]. Ultrasound-guided approach PNB was assumed to achieve more rapid onset, better blockade quality, and higher success rate than the anatomical approach or nerve stimulation guidance [56,57]. The duration of single injection nerve block is variable, but the effective time is usually about 12 to 24 h. In our study, neurological symptoms were followed up on 1 day after surgery. Transient sensory and motor deficits in the involved extremity were noted on 1 day after surgery in both groups, however, it showed no significant difference to the control group. We supposed that neurological impairments might not relate to PNB, but resulted from preexisting pathology such as tissue trauma. All participants resolved their neurological deficits a few days before discharge.

There were some limitations in this study. In the study, we enrolled included upper and lower extremities, not a single procedure. Different types of peripheral nerve blocks were performed depending on operation sites. The study was not large enough to measure the safety of PNB under general anesthesia and to confirm the protective effect of PNB on surgical-induced stress response. Lack of blinding (PNB/no PNB for the patients), short length of evaluation, and only NRS after PACU were also major limitations in our study. Finally, additional nociception monitors, such as analgesia nociception index, might be included when PNB is used during general anaesthesia to detect early block failure [58]. Future large-scale and long-term follow-up research should be undertaken.

## 5. Conclusions

The combination of preemptive ultrasound guided PNB under general anesthesia provides patients comfortable, amnesia, and immobile. It might be a safe alternative to minimize surgical-induced stress by attenuating IL-6 level, reduces postoperative pain intensity, and improve post-anesthesia recovery profiles. PNB is considered as an adjuvant to general anesthesia, as it offers several advantages over general anesthesia alone during extremity surgery if not contraindication.

## Figures and Tables

**Table 1:** The patient characteristics between both groups. The level of significance was set as *p* values < 0.05.

	Group N (*n* = 27)	Group C (*n* = 25)	*p* value
**Age (year)**	60.23 ± 14.37	51.56 ± 14.46	0.053
**Gender (Male/Female) (*n*)**	8/19	12/13	0.256
**Weight (kg)**	67.59 ± 12.25	66.70 ± 13.30	0.758
**Height (cm)**	158.33 ± 9.37	160.70 ± 10.64	0.550
**ASA ^1^ class I/II/III (*n*)**	0/9/18	0/11/14	0.321
**Surgical procedures** Fracture(*n*)/Arthroplasty(*n*)	18/9	16/9	0.84
**Surgical sites**			0.629
Upper limb, *n* (%) Lower limb, *n* (%)	7 (25.9%) 20 (70.1%)	8 (32.0%) 17 (68.0%)	
**Propofol dose (mg)**	1124.85 ± 471.31	1093.48 ± 410.56	0.819
**Operation period (min)**	127.33 ± 48.33	116.00 ± 44.21	0.373

^1^ ASA: American Society of Anesthesiologists. Continuous data (age, weight, height, propofol dose, operation period) were expressed as mean ± SD. Categorical data (ASA class, gender, surgical procedures or sites) were expressed as *n* or *n*(%). *p* values < 0.05.

**Table 2:** Analgesia and recovery profile after anesthesia in the post-anesthesia care unit (PACU). The level of significance was set as *p* values < 0.05.

	Group N (*n* = 27)	Group C (*n* = 25)	*p* value
**Peak verbal NRS ^1^ pain score (0-10)**	1.15 ± 1.81	5.40 ± 2.48	0.001 *
**Morphine consumption (mg)**	1.85 ± 2.04	6.44 ± 3.22	0.001 *
**Rescue analgesics ^2^** **,** ***n*** **(%)**	3 (11.1%)	14 (56.0%)	0.003 *
**Wake-up time (min)**	17.04 ± 10.49	26.60 ± 15.32	0.025 *

^1^ NRS = Numeric Rating Scale. ^2^ Postoperatively, regular administration of intravenous morphine 0.05 mg/kg if NRS greater than 3 in the PACU. When the patients had poor response to morphine in the PACU, the following rescue analgesics were administered according to the anesthesiologist’s preferences to improve pain control: Parecoxib or ketorolac. Continuous data (peak verbal NRS ^1^ pain score, morphine consumption, and wake-up time) were expressed as mean ± SD. Categorical data (rescue analgesics) were expressed as *n*(%). * *p* values < 0.05.

**Table 3:** Changes of serum interleukin 6 concentration during surgery. The level of significance was set as *p* values < 0.05.

	Group N (*n* = 27)	Group C (*n* = 25)	*p*-value
Pre-incision (pg/mL)	6.04 ± 4.92	5.97 ± 5.44	0.684
Incision 1 h (pg/mL)	3.79 ± 4. 21	5.19 ± 5.93	0.437
Incision 2 h (pg/mL)	5.36 ± 5.01	12.95 ± 15.32	0.027 *

All data were expressed as mean ± standard deviation. * *p* values < 0.05.

**Table 4:** Adverse events, sensory/motor deficit and satisfaction on the post-operative day 1.

	Group N (*n* = 27)	Group C (*n* = 25)	*p* value
**Adverse events**			
Dizziness, *n*(/%)	2 (7.4%)	8 (32%)	0.024 *
PONV ^1^, *n*(/%)	0 (0%)	3 (12%)	0.141
Pruritus, *n*(/%)	0 (0%)	0 (0%)	1
Respiratory episode ^2^, *n*(/%)	0 (0%)	3 (12%)	0.064
**Sensory blockade ^3^ (0/1/2) (*n*)**	26/1/0	25/0/0	0.130
**Motor blockade ^4^ (0/1/2) (*n*)**	23/4/0	23/2/0	0.187
**Satisfaction (1/2/3/4) ^5^ (*n*)**	4/21/2/0	0/22/3/0	0.365

^1^ PONV = post-operative nausea and vomiting. ^2^ Respiratory episode was defined as oxygen saturation (spO2) below 90% and oxygen supply was needed. ^3^ Sensory block scoring as: 0 = no sensory block, 1 = loss to cold sensation and no loss to pinprick sensation, 2 = loss to both cold and pinprick sensation. ^4^ Motor block scoring as: 0 = No motor block, 1 = Partial block, 2 = Complete motor block. ^5^ Satisfaction grading as: 1 = very satisfied, 2 = satisfied, 3 = fair, 4= unsatisfied. * *p* values < 0.05.
